# Applying Value Sensitive Design (VSD) to Wind Turbines and Wind Parks: An Exploration

**DOI:** 10.1007/s11948-014-9536-x

**Published:** 2014-04-18

**Authors:** Ilse Oosterlaken

**Affiliations:** Section Economics of Technology and Innovation; Faculty of Technology, Policy and Management, Delft University of Technology, P.O. Box 5015, 2600 GA Delft, The Netherlands

**Keywords:** Aesthetics, Ecology, Justice, Community acceptance, Social acceptance

## Abstract

Community acceptance still remains a challenge for wind energy projects. The most popular explanation for local opposition, the Not in My Backyard effect, has received fierce criticism in the past decade. Critics argue that opposition is not merely a matter of selfishness or ignorance, but that moral, ecological and aesthetic values play an important role. In order to better take such values into account, a more bottom-up, participatory decision process is usually proposed. Research on this topic focusses on either stakeholder motivations/attitudes, or their behavior during project implementation. This paper proposes a third research focus, namely the ‘objects’ which elicit certain behavioral responses and attitudes—the wind turbine and parks. More concretely, this paper explores Value Sensitive Design (VSD) as way to arrive at wind turbines and parks that better embed or reflect key values. After a critical discussion of the notion of acceptance versus acceptability and support, the paper discusses existing literature on ecology and aesthetics in relation to wind turbine/park design, which could serve as ‘building blocks’ of a more integral VSD approach of the topic. It also discusses the challenge of demarcating wind park projects as VSD projects. A further challenge is that VSD has been applied mainly at the level of technical artifacts, whereas wind parks can best be conceptualized as socio-technical system. This new application would therefore expand the current practice of VSD, and may as a consequence also lead to interesting new insights for the VSD community. The paper concludes that such an outcome-oriented approach of wind turbines and park is worth exploring further, as a supplement to rather than a replacement of the process-oriented approach that is promoted by the current literature on community acceptance of wind parks.

## Introduction

The large-scale introduction of onshore and offshore wind turbines remains a societal challenge, despite its potential for providing sustainable energy. In the Netherlands and elsewhere issues of social acceptance have meant that offshore wind energy is increasingly being considered as an option, despite greater technical and economic difficulties as compared to onshore wind. However, so Hagget (2011, p. 503) points out, “the first offshore wind farms—in the UK and elsewhere around the world—have not been free from opposition.” Many social acceptance issues for onshore wind also apply to offshore wind (Wolsink 2010), although “maybe with slightly different characteristics than for onshore” (Huber and Horbaty 2010, p. 29). Considering political goals to increase sustainable energy production, it is not surprising that this issue of social acceptance of wind energy has received a lot of attention, both in practice and from researchers. In a seminal article on renewable energy innovation Wüstenhagen et al. (2007) identified three interrelated types of social acceptance: socio-political acceptance (of wind energy in general by politicians, policy makers and citizens), market acceptance (by e.g. electricity firms and investors), and community acceptance (by stakeholders, of concrete wind energy projects). This paper will focus on the latter.

This paper proposes that the adoption of Value Sensitive Design or VSD may be helpful to achieve a responsible, socially acceptable implementation of wind energy. VSD is an approach that originates from the field of ICT, but that is increasingly being applied to other technologies (Van den Hoven et al. forthcoming). VSD is based on an assumption that the configuration of technology is not value-neutral, and that generally different alternatives exist, which can and should be compared and assessed against relevant values (Van de Poel 2009). It therefore aims to pro-actively take values into account throughout the design process. Any VSD project will of course have some specific object of design. As ‘wind energy’ is rather broad, a range of design projects could be distinguished within this domain. One could think of wind turbine components, wind turbines, complete wind parks, storage facilities, HVDC converter stations, transmission networks, the integration of large-scale offshore wind parks into the electricity net, or even the electricity system as a whole—for example, a European super grid or smart grids enabling the large-scale integration of intermittent, renewable energy sources like wind energy. All of them may in principle raise certain value issues, possibly making VSD a sensible approach. However, at the moment both value and social acceptance issues are especially salient in smart grids and wind parks. For example, smart grids raise issues of privacy, security and reliability, for which VSD may offer a partial solution.^1^ This paper focusses on wind parks, and as a derivative also on wind turbines—as these are obviously the defining element of wind parks.

The application of Value Sensitive Design to wind parks and turbines could arguably contribute to solutions that are more *acceptable* from the perspective of relevant values such as justice, sustainability, and well-being. That implementing this idea in practice could contribute to a larger degree of actual social *acceptance* is merely a hypothesis at this moment, the proof of which is beyond the scope of this paper. The paper will also not engage in evaluating concrete turbine or wind park designs from a value perspective, or investigate any actual design processes in this domain. Rather, the paper takes a step back and provides an in-depth exploration of the idea of applying VSD to wind parks and turbines, informed by different bodies of literature. One key issue is that wind parks may be best understood as socio-technical systems, whereas VSD has traditionally focused on technical artifacts. Another, somewhat related, key issue is the demarcation wind park projects as feasible VSD projects. This new application would therefore expand the current practice of VSD, and may as a consequence also lead to interesting new insights for the VSD community.

The structure of the paper is as follows. It will first discuss the importance of values in community acceptance of wind parks. After a brief critical reflection on acceptance—contrasting it with both acceptability and support—the VSD approach will be introduced. Next, I will discuss what leads and building blocks for a VSD approach are being offered in the current literature on wind energy. This sets the stage then for a discussion of the two key issues or challenges mentioned above. The paper will end with some conclusions.

## The Importance of Values in Wind Park Community Acceptance

According to the well-known NIMBY (“Not in My Backyard”) explanation, local residents reject a wind project in their geographical vicinity because they are trying to maximize individual utility, despite them having—in recognition of the common good—a positive attitude towards wind energy in general. It is thus, just like the prisoner’s dilemma and the tragedy of the commons, a specific form of a social dilemma. Invoking the NIMBY explanation is often accompanied by authorities and experts judging local people to be ignorant, irrational or selfish. The solution is generally sought in providing people with more knowledge or information about why the project would be beneficial to society or why the risks would be acceptable, or in introducing more strict top-down planning procedures—ignoring the real arguments at stake (Wolsink 2006). In the past decade or so, however, a range of studies has quite forcefully criticized the NIMBY explanation (see e.g. Wolsink 2006; Haggett 2011), by showing that people often (also) have non-selfish and more complex reasons for their opposition. This opposition is often closely tied up with moral or public values. As Kempton et al. (2005 p. 124) put it:

We have three reasons for our not using this term [NIMBY]. First, it is generally used as a pejorative implying selfishness as an underlying cause; second, it appears to incorrectly describe much local opposition to wind projects; and third, the actual causes of opposition are obscured, not explained, by the label.

In this section I will discuss some of the many research findings that point towards the importance of various kinds of values for the explanation of either opposition to or acceptance of wind energy projects.^2^

Wolsink (2000), for example, concludes from a survey in the US and the Netherlands that “most people with [alleged] NIMBY-feelings are not so much in favor of wind power at all” (p. 54), and that “the strongest impact on the [general] attitude [towards wind power] concerned the aesthetic value of wind turbines (p. 51).”^3^ The importance of aesthetics is confirmed by the results of a study of Ladenburg and Dubgaard (2007, p. 4068/69) in Denmark, which “strongly indicate that even if a large proportion of respondents (and people in general) are unable to see offshore wind farms on a daily basis [because there are none within sight from their residence or summer house], the visual disamenities are still perceived as being important.” This is indicated by their “willingness to pay” for “siting wind farms further offshore to reduce the visual disamenities.” And for those who do have a positive general attitude towards wind power, says Wolsink (2000) “the decision to support or oppose such a [concrete] project will depend primarily on the visual quality of the [selected] site” (p. 51). Either way, contra NIMBY, “the personal assessment of the benefits of wind power hardly enters the argument” (p. 56). It is rather aesthetic values held by people that seem to be crucial.

Furthermore, the NIMBY explanation—ascribing selfish motives to people—seems to completely ignore the possibility that local opposition may actually be based on a plausible claim of injustice taking place. Overall societal cost-benefit analyses tend to ignore the question whether the benefits and the costs or risks of an initiative are fairly distributed over different groups in society (distributive justice), whereas this is actually an important ethical issue for many new technologies or technological projects (Asveld and Roeser 2009). Indeed, so Wolsink (2007, p. 1188) concludes for wind power implementation more specifically, “feelings about equity and fairness appear the determinants of ‘backyard’ motives, instead of selfishness.” Perceptions of fairness, says Wolsink (2007, p. 1203), are amongst others “strongly connected with […] core values about how society should take such decisions, not only within the public, but among all stakeholders involved in such processes” (procedural justice).

Based on seven Australian wind farm cases and using grounded theory, Hall et al. (2013) recently concluded that the stakeholder concerns with the most impact on social acceptance were related to four value themes: trust, distributive justice, procedural justice, and place attachment. Distributive justice and procedural justice were already mentioned. Trust is closely connected to procedural justice, and to values such as honesty and transparency. The last one, place attachment, is relevant for people’s assessment of visual changes to a place or landscape—so for people’s aesthetic evaluation. In a UK offshore case study Devine-Wright and Howes (2010) found that the degree to which people’s ‘attachment to place’ leads to opposition depends on whether the project is situated in an area that is considered to be of great natural beauty, or in an area that is already industrialized or in decline. This is in line with the before mentioned finding of Wolsink (2000) that the quality of the project site is an important factor. Again, opposition is thus not a matter of people simply trying to maximize individual utility—although it may of course be that some people are actually ‘NIMBY’s’ (Bell et al. 2013).

## ‘Support’ and ‘Acceptability’? A Critique of the Notion of ‘Acceptance’

The literature, so it was shown in the previous section, highlights a range of values as particularly important for the social acceptance of wind energy projects. Before discussing VSD as one way to pro-actively address value issues in wind energy, I will reflect briefly on the notion of ‘acceptance’ itself—a reflection intended to lend further support to the exploration of alternative approaches to implementing wind energy.

Firstly, I would like to draw attention to an article by Batel et al. (2013). They point out that the focus on social acceptance, and a certain interpretation of this concept, has been taken for granted in the renewable energy literature. They present some empirical evidence that a distinction between acceptance and support exists.^4^ Acceptance, so they explain, is a passive reaction to something which is proposed externally, and the absence of active opposition against something is generally taken as a sign of acceptance. Support, on the other hand, is a more action-oriented response, where people actually approve of something and are willing to defend or promote something. It implies “agency *for* and engagement with something” (p. 2). They speculate that a narrow focus on merely acceptance “might prevent the sustainability of these technologies in the long term” (p. 4), because it could—just like the NIMBY concept—contribute to maintaining and legitimizing a top-down planning approach. To this I would like to add that a narrow focus on acceptance could also encourage a narrow view on what values need to be taken into account, and in which way this needs to be done—namely focused on what seems most instrumentally efficient in creating such acceptance in the short term. Long term support, however, might require taking a very broad range of values pro-actively and more seriously into account.

Secondly, whereas Batel et al. contrast acceptance with support, Cowell et al. (2011) contrast acceptance with acceptability. Their focus is on the value of distributive justice, and the practice of developers to offer community financial or economic benefits in order to create local acceptance. “Care must be taken”, they say, “not to elide *ex ante* acceptability with *ex post* acceptance” (p. 553/4)—noticing that “once a wind farm has been completed, people find ways to *accept* it, as indeed people accept all sorts of unwanted outcomes […] and no longer actively resist the state of affairs” (p. 553). Furthermore, they say, apparent acceptance—in terms of lacking effective resistance—may also be a consequence of people anyways feeling/being powerless in the face of politics and societal change. There are indeed indications that the siting of renewable energy facilities tends to concentrate in areas that have the most vulnerable and marginalized communities. Blowers (2010, p. 169), looking at nuclear plant siting in the UK, concludes that the process “imposes a burden of risk on peripheral communities, least able to resist, offered neither compensation nor effective participation in decision making.” Similarly, Van der Horst and Toke (2010, p. 214), looking at wind farm siting in rural England, draw attention to “the strong significance of local democratic deficit (i.e. low voter turn-out) as a predictor of a ‘positive’ planning outcome.” In short, what is at stake is not mere acceptance, but the ethical question of acceptability.

These two different ways of critiquing the focus on acceptance seem to supplement each other, and an interesting hypothesis for further research would be that a way to create long-term *support* for wind farms is not merely a more participatory process, but ensuring *acceptability* of the outcome in terms of key values.

## Process Versus Outcome Orientation

If one accepts that values are salient for wind energy implementation, the question is then how to take them into account better. In principle it seems that there are two different approaches to doing so. The first is improving the *process* of decision making at different levels, so that it reflects or incorporates relevant values better. The value of procedural justice is of course closely connected to this process-oriented approach, and also the value of trust comes into play here. Acknowledging the importance of values, Wolsink (2000, 2007) for example argues for more bottom-up, participatory, collaborative planning and decision-making arrangements with respect to wind energy. Indeed “frequently, participation is promoted within the [wind energy] literature as a tool with which to ensure greater public acceptance” (Aitken 2010 p. 1839).^5^ According to Jobert et al. (2007, p. 2752) “two general [research] approaches to the issue of social acceptance can be identified” in the literature on wind energy. The first is “orientated towards public opinion (global and local), working with opinion polls or discussion groups to identify the *motivations and attitudes* of the public” (emphasis is mine). And the second “analyses how a project or a program is constructed to understand why it is accepted or rejected, focusing either on public policy or on *actors’ behavior* *during the implementation*” (emphasis is again mine). Both these types of research can provide valuable knowledge in support of this process-oriented approach to taking values seriously.

It seems then that the scholarly literature on the social acceptance of wind energy tends to ‘black box’ the materiality of wind parks and turbines, as design considerations and design alternatives/solutions have not received any systematic attention. This realization opens up avenues for a third research approach, one which is focused on the ‘objects’ towards which public motivation/attitudes are directed, the ‘objects’ which elicit certain behavioral responses in the process of their development: the wind parks and turbines themselves. Such research could be supportive of a second way to taking values more seriously into account in practice, namely making sure that *outcomes*—wind parks and turbines—better reflect these values. This paper proposes Value Sensitive Design (VSD), being a pro-active approach to taking values systematically into account during the design phase, as a way to make this outcome-oriented approach more tangible and concrete. The next section will discuss VSD in more detail, but the general idea is that relevant values—such as distributive justice or well-being—should become embedded in new designs through systematic reflection on them during the full process of design.

The proposal made here is not that such an outcome-oriented approach should replace a process-oriented approach. Both approaches are not mutually exclusive, but should rather be seen as interconnected^6^ and complementary. One can for example argue that the *process* matters both as an end in itself (i.e. as an expression of values like democracy, respecting people’s agency, procedural justice), and as a means towards a more high-quality, value-sensitive outcome—as stakeholders may e.g. be able to shed new light on relevant values and their meaning in a certain context. Furthermore, so Aitken (2010 p. 1839) claims, “meaningful participation must empower participants and facilitate *relevant and sustainable* *outcomes*” (emphasis is mine). Indeed, the design outcome is where relevant values become embedded and materialized in more or less comprehensive and suitable ways. It is the existence of realistic design alternatives that are substantially different from a value perspective that makes a deliberative, participatory decision process truly meaningful. If no such alternatives would exist and be feasible, such a process would be vacuous and redundant in an important sense. In addition one could argue that even if the conditions for making a process more fair or just are met, there is still no guarantee that the outcome of such a local process is per definition just. The outcome arguably also needs to be assessed against some wider moral standards. This is probably most obvious for moral standards or principles related to intergenerational justice; Future generations are per definition not participating in the process, even though their interests matter from a moral perspective.

## Value Sensitive Design

VSD takes, according to Friedman and Kahn (2003, p. 1178), an “interactional” position on the question of “how exactly […] values become implicated in technological design.” This position means taking a middle road between technological and social determinism, acknowledging that both design features and usage or application in a certain context matter. A distinguishing feature of VSD is that it does not focus on a single value—such as e.g. ‘design for sustainability’ or ‘privacy by design’ does—but rather provides a general overarching framework to address a range of values throughout the design process. Its basic approach is that an iterative, tripartite process is needed in which conceptual, technical and empirical investigations are being integrated (Friedman et al. 2001; 2006). The conceptual phase concerns “philosophically informed analyses of the central constructs and issues under investigation” (Friedman et al. 2001, p. 2). Key questions include which values are relevant, how they should be understood (what do we mean with e.g. well-being or distributive justice?), and which trade-offs between conflicting values are acceptable (is lowering safety levels acceptable for achieving sustainability?). The technical investigation looks into the question “how existing technological properties and underlying mechanisms support or hinder human values” and involves “the proactive design of systems to support values identified in the conceptual investigation” (Friedman et al. 2001, p. 3). Empirical investigations, finally, complement conceptual and technical investigations. Examples are research into aspect of the context of implementation that co-determine to what degree values will in the end be realized, and stakeholder research into people’s perception of relevant values and their proper conceptualization. It has also been proposed that VSD always includes the activities of the discovery, translation and verification of values (Flanagan et al. 2008). Van de Poel (2013) discusses the activity of translation in more detail. He proposes using a “value hierarchy” of three levels: *values* need to be translated into *norms*, which in turn have to be translated into *design requirements.* 7 In practice a design may or may not realize the intended values, hence verification is needed.

The literature on VSD includes both cases studies (e.g. Cummings 2006; van Wynsberghe 2013) and more general reflections and discussions. One thing that has been discussed is its relationship with participatory design (PD). Borning and Muller (2012, p. 1130) “suggest that the traditional PD commitments to co-design and power sharing be carefully considered in VSD projects as well,” and that VSD may benefits from developments which have taken place in the PD literature. Manders-Huits (2011, p. 271), for example, voices the critique that “VSD does not have a clear methodology for identifying stakeholders”, although Borning and Muller (2012, p. 1130) would emphasize the importance of “giving voice to the participants in the VSD study rather than prescribing particular methods.” According to Friedman and Kahn (2003, p. 1183) VSD takes “a middle ground” in the debate around the universality or cultural relativity of values, “one that allows for an analysis of universal moral values, as well as allowing for these values to play out differently in a particular culture at a particular moment in time.” This is criticized by Manders-Huits (2011, p. 271), who finds it problematic that “the concept of values, as well as their realization, is left undetermined” and that “VSD lacks a complimentary or explicit ethical theory for dealing with value trade-offs”. Contrary to that, Borning and Muller (2012, p. 1127) feel that these are the sort of issues that “VSD as such simply doesn’t need to take a position on”, as “this doesn’t help advance the development and application of VSD”, and may even impede it as people may dislike the specific answer given. In short, the idea of VSD still gives room to different concrete methods or normative background positions.

The VSD approach has so far not been explicitly applied to wind energy, but two examples that do not concern wind energy, yet are in some respect relevant, can illustrate the idea of VSD and why it may be fruitful to explore a wind park application of VSD. The first example concerns the Eastern Scheldt Storm Surge Barrier, which like offshore wind parks concerns the introduction of a major engineering work in a coastal area. This storm surge barrier was built in the 1970s in the Dutch province of Zeeland, as a response to a massive flood disaster which took place in 1953. After the flooding took place, a Delta plan was initially made that included closing off the Eastern Scheldt estuary. This led, however, to a lot of opposition from environmental organizations, who pointed out that a valuable and unique ecological area would be lost if salty sea water and tides would no longer be part of the ecosystem. This conflict only became resolved when a proposal was developed to build a storm surge barrier instead, which is a barrier that is normally open, but can be closed when conditions are such that there is a risk of dangerous flooding. This design solution was “a creative compromise to balance the two moral values, safety and ecological care, that were at stake” (Van de Poel and Royakkers 2011, p. 169). Even though this was at the time not conceptualized as VSD, the example illustrates how clever design may at least sometimes be able to solve a value conflict that was previously dividing people on the best way to deal with a societal challenge.

The second example concerns the design of nuclear energy plants. A major decision for any plant design is whether the reactors should make use of a closed or an open fuel cycle. From an engineering perspective this choice depends on one’s assessment of the alternatives on criteria like cost, reliability, and efficiency. Research by Taebi and Kloosterman (2008, forthcoming) has shown, however, that these two fuel cycles are also very different from the perspective of the values of intergenerational justice, public health and safety, security and sustainability. Unfortunately it is not the case that one of the alternatives scores better on all these values, so that the choice means in effect prioritizing certain values over others. The idea of VSD is that such moral deliberation should be made explicit throughout the design process. Although this example can be taken to illustrate the idea behind VSD, it can also be taken to show that VSD is no panacea for all social acceptance issues. It is, after all, reasonable to reject both design alternatives for nuclear reactors by arguing that society should opt for other energy options instead—be it wind energy or something else. In fact, as every energy option will have some negative consequences, it does not suffice to weigh risks and benefits of a single energy technology, or try and optimize it; rather what is needed is evaluating complete energy scenarios (Dumke and Hillerbrand 2014). Yet even when acknowledging this, it makes sense to also investigate the possibilities for value-sensitive design of specific energy technologies, so that the best possible design alternative can be taken into account in our scenarios and subsequent deliberations.

## Aesthetics and Ecology in Wind Turbine and Park Design

The previous section claimed that VSD has so far not been applied to wind energy. This seems to be true if one considers VSD as an approach advocated by a specific body of literature and with certain specific characteristics, such as always taking a wide range of values into account, and making use of the tripartite approach described before. Yet it is certainly not true that specific values, such as for example ecological or aesthetical values, have never before been explicitly considered in relation to wind turbine and wind park design.

Concerning ecology, some examples have been included in a recent report on best practices in wind energy by the International Energy Agency (Huber and Horbaty 2013). It refers, for example, to a “nature inclusive design” process that has been developed in the Netherlands. Part of this approach is that “nature-development is planned in the same area [as wind turbines] and operated as one project. In consequence, the total effect of the project might be positive for nature.” The authors illustrate this approach with two Dutch projects, one near shore project in which “a ramp to safeguard the farm from collisions with ships is built in such a way that it will serve as a refuge for birds”, and a project in which “turbines are built onshore on a dam. Within the wind farm project, an extra dam on the seashore was built as a nesting and refuge place for seagulls” (p. 18). In this way both projects achieved a positive effect on wildlife, which apparently contributed to respectively planning permission and extra support for the project in question. Another example can be found in a book by Beurskens (2011), reporting on the Dutch We@Sea research program (2004–2010) on offshore wind energy. It mentions an alternative, environmentally friendly monopole foundation which was developed for offshore wind turbines. This included research into the installation method, as monopole ramming normally causes a very sharp and intensive underwater sound, which is carried over long distances and which may harm the hearing ability of sea mammals and fish larvae. Still, gravity foundations, which do not require piling operations, might be even better if one would like to minimize wildlife disturbance (Kondili and Kaldellis 2012). And in France attempts have been made to develop and design wind parks in such a way that they lead to a bird-friendly landscape (Nadaï and Labussière 2010).

Aesthetics is amongst others discussed by Gipe (1993, 2002), who presents some concrete guidelines for how to design wind parks in such a way as to “minimize visual impact.” According to him the “single, most important consideration” for designing wind parks is “providing visual ‘unity’ in type of turbine, tower and spacing” (Gipe 1993, p. 245). Sharpe (2011) discusses the importance of aesthetics and visual impact for a specific type of wind power application, namely single wind turbines applied in urban environments, integrated in or attached to buildings. The same type of wind power application is also being investigated by a group at Penn State University, which combines “technical, environmental and aesthetic research and design studies.”^8^ An extensive treatment of the landscape aspects and aesthetics of wind turbine/park design can be found in a Dutch report by Schöne (2007). According to Schöne the visual effects of the youngest generation of wind parks is substantially different from the older wind parks with smaller turbines. He discusses many different aspects, such as the type of landscape, the micro-siting of the turbines and the design of the turbines themselves. Five ideas for a new way of looking at large-scale wind park design in relation to the surrounding landscape are discussed in an earlier publication by the same author (Schöne 2004).

This existing literature on ecology and aesthetics in relation to wind turbine and wind park design could be seen as providing building blocks for a more systematic VSD approach of wind energy in the future. A key reason to advocate such an approach is that this would enable identifying and investigating value conflicts and trade-offs, so that design decisions can be made in a transparent way. The examples of the design of nuclear reactors and of the Eastern Scheldt Storm Surge Barrier already illustrated why this is considered important in VSD. That this integral consideration of a range of values may also be desirable for wind turbine/parks design can be shown by the following example of aesthetics and ecology pointing in a different direction:

The increased height of a wind turbine [leading to higher nominal power per turbine] means that the possible impacts of the turbine become more intensive, such as the visibility of the turbine from places of special interest, like archeological sites, tourist destinations and so on. […] Generally, in sites with natural beauty and special esthetic, the installation of smaller wind turbines can be characterized as a secure selection, capable to protect the wind park project’s implementation from several [social acceptance] problems. On the other hand, the installation of a large number of wind turbines of lower nominal power [so low turbines] instead of few wind turbines of higher nominal power [so high turbines] increases the probability of birds’ collisions with the wind turbines’ spinning blades. […] Ornithologists [therefore] suggest the installation of [a] few [high] wind turbines of higher nominal power in large distances between them, in order to approach the total wind park’s nominal power (Al Katsaprakakis and Christakis 2012, p. 189).^9^

For reasons of efficiency larger distances often accompany the choice for higher wind turbines, as higher nominal power increases the distance needed between wind turbines to prevent “wake” effects between different turbines. Ornithologists, according to Al Katsaprakakis and Christakis (2012), apparently also recommend larger distances between wind mills. However large distances may—just like high wind turbines—be undesirable from an aesthetic perspective, as concentration (Schöne 2007) and creating visual unity (Gipe 2002) are important for diminishing visual impact. Wind turbine height seems furthermore relevant in relation to another value, namely human well-being: “aerodynamic noise can […] be minimized by reducing the operating speed”, and “due to the larger rotor size, bigger turbines are designed to run slower to keep the optimal tip speed ratio” (Mathew and Philip 2012). If such noise indeed negatively affects human well-being, this provides a reason to prefer high turbines. This short discussion is not meant to defend a particular design, but rather to illustrate that in designing a wind park explicit ethical deliberation needs to take place on how to balance values like human well-being, aesthetic pleasantness, ecological integrity, and distributive justice.^10^

An objection that one may have to the feasibility of applying VSD to wind turbines is the problem of cost-effectiveness. Cost-effectiveness is actually the main reason why wind turbines have tended to get bigger and bigger—it is still an important challenge for wind energy, although nowadays more for far-offshore than for near-shore or onshore wind parks. One might reply that cost-effectiveness seems like a very mundane consideration that should not override important value considerations. However, it might also be argued that cost-effectiveness—despite perhaps being (somewhat) at the expense of bird and landscape protection—is a requirement for achieving socio-political acceptance of a renewable energy source like wind energy, which is in turn needed to realize the value of inter-generational justice within our energy-intensive society. Whether any of these arguments make sense is partly an empirical question; For example, how important are cost considerations for creating socio-political acceptance in either the short and the long term? How big is the actual impact of certain turbines on wildlife? In that sense moral deliberation, and therefore also VSD as the context in which such deliberation takes place, could benefit from “empirical investigations”—although a moral conclusion cannot straightforwardly be drawn from any such facts.

With respect to the economic feasibility of the VSD of wind turbines it may also be objected that “to lower the cost of wind power still further takes mass production of turbines” (Wizelius 2007, p. 4). Considering economic realities it thus does not seem feasible to have a bespoke turbine design for each wind park. Yet “most manufacturers offer several models, with different hub heights and/or rotor diameters, so the turbines can be tailor-made for specific sites” (Wizelius 2007, p .75)—or at least to some degree. And it is not inconceivable that the problem of social acceptance in combination with the idea of VSD encourages the further development of alternative designs based on value considerations. Or at the least encourages a better articulation of the value-laden choices made in current turbine designs—which in turn would facilitate a more value sensitive turbine choice when developing a wind park. In short, from the perspective of wind park design, the issue seems to be more a matter of value-sensitive turbine *choice*, than of value sensitive turbine *design.*

## Demarcating Wind Parks as VSD Projects

Whereas the emphasis in the previous section was on wind turbine design, this section will focus on the possibility of a VSD approach to wind park design. This possibility raises questions about the boundaries and scope of such design projects. I will subsequently discuss location choice, multi-space usage, and overall project set-up. *Location choice* is, as we also saw before, a major factor in community acceptance (Wolsink 2010)—and value-sensitivity may therefore be crucial in location choice. Whether location choice is also part of the design challenge depends. From a value perspective arriving at the best solution for an energy need may very well not be about a choice between one design or another, but about a choice between one design at one location, and another design at another location. However as planning procedures for wind farms are in general quite lengthy and costly, it may in practice not be realistic to expect a project developer to simultaneously look into several different combinations of design and location. Of course the design for a certain location will always partly depend on the features of that location.

Another demarcation issue in wind park design is whether the project should concern merely the design of a wind farm, or whether the design should facilitate a *multi*-*sector usage* of the space involved. This question is specifically relevant for offshore wind farms, where competing usages include recreation, fishery, (naval) transport and gas- and oil exploration. There have, for example, been proposals for technically integrating wind power production with offshore gas exploitation—the so-called ‘super wind concept’ (Hemmes et al. 2008). Another idea is that solar PV and/or wave energy converters are integrated with the supporting construction of the wind turbines (Marquis et al. 2012). The integration of marine aquaculture or fish farms within wind parks is another possibility—one which may provide a solution for conflicts of interest between the fishery industry and wind farm developers (see Gray et al. 2005). Despite the existence of such ideas, multifunctional concepts for the design of offshore wind parks are currently, however, hardly systematically studied or even implemented.^11^ One reason may be that such multi-sector usage of wind parks still faces many practical obstacles, such as a lacking legal framework (Michler-Cieluch et al. 2009). A major reason for wanting to expand the scope of the design of offshore wind parks in such ways is that this enables cost sharing—as mentioned, making large and far offshore wind farms financially viable is still a challenge. Nevertheless multifunctional wind parks may also contribute to the social acceptance of offshore wind, as it allows taking into consideration competing claims for the usage of space. Enlarging the scope of design in this way means allowing for non-conventional, innovative solutions, thereby increasing the solution space.^12^ This may in turn result in being able to accommodate or respect relevant values to a larger degree—just as in the case of the innovative solution for the Eastern Scheldt Storm Surge Barrier.

A further question that one may ask about the scope of wind park design is whether it should, in addition to its physical and technical design, also include the overall project set-up—such as its form of ownership, or monetary schemes connected to the wind park. Widening the scope of wind park design to include such aspects would—just as with multi-space usage—increase the solution space and therefore possibly the range of values that can simultaneously be accommodated—especially distributive justice. Providing community benefits is, for example, one possible way to increase the acceptability of a project.^13^ It does however raise questions with a moral component,^14^ and explicit moral deliberation seems to be desirable as certain ways of providing community benefits may be more problematic than others. Cowell et al. (2011), for example, mention a case where local people preferred getting free electricity during the period that the wind turbines were in use. “Given that making electricity free might do little to reduce consumption”, they notice (p. 552), “one can see here the tension between a rationale for community benefits that prioritizes satisfying local communities, and a rationale that favors long-term environmental sustainability.” Thus explicit moral deliberation is required when choosing a solution.

Interestingly, the specific options that are feasible and the solution finally chosen will partly depend on or be influenced by the broader institutional environment. Regarding the provision of community benefits Cowell et al. (2011) argue that

the nature of such benefit streams reflect wider institutional characteristics of renewable energy provision in those countries: thus in Denmark and parts of Germany, ‘community benefits’ arise mainly from cooperative and farmer ownership of turbines; in France, from increased local tax revenues attendant on designating wind energy development zones; and in Spain from company agreements to invest in the regional economy. In the UK, the typical form of community benefit arises where a major, commercial energy developer offers a fund, per annum, per megawatt of installed capacity, to community organisations, for spending on local projects (p. 540).

VSD processes may thus be shaped by the wider institutional arrangements, and they should also be studied from this perspective. This may lead to the conclusion that a re-design of such institutions, so in a sense VSD at a meta-level, might be desirable to expand the set of alternatives available in the process of the VSD of wind farms, or to increase the chances of the adoption of alternatives that seem more desirable from a value perspective.

One thing that would be interesting for future research is integral, in-depth case studies of different concrete design alternatives which were considered during wind park development processes. Such case studies could contribute to making this idea of VSD of wind parks more concrete and tangible. These case studies could look at e.g. the turbines involved and the micro-siting, but also at how the institutional setting constrained or facilitated the design process and/or feasible design alternatives. Such cases should address how different design alternatives were/could/should be judged from the perspective of different values—so the reasoning involved in linking values to design proposals. Such case studies could provide content for some sort of ‘design library’, which would collect and make accessible a wide range of different design alternatives, and their evaluation from different value perspectives. This design library may become a helpful source of inspiration and information in developing new parks and choosing turbines for it.

A wind park project that could, for example, make an interesting case is Zuidlob,^15^ one of the biggest onshore wind parks in the Netherlands. It was developed by energy company Nuon, together with 63 agrarian companies in the area. The last of 36 turbines was installed in March 2013. The project paid a lot of attention to the distribution of costs (e.g. shadows, effects on real estate prices) and benefits over the participating farmers—so to distributive justice. As the micro-siting of the wind turbines was considered important for this, ten different spatial designs were seriously taken into consideration. Crucial in the process was that that before the micro-siting design was finalized, an agreement was reached amongst participants on ‘rules’ that would lead to a fair distribution of benefits. For example, the compensation for somebody who would get a windmill on his land, the compensation per kilometer of road over somebody’s land, etc. Such rules can subsequently become input for an evaluation of different design options. The project developers also wanted to keep their options with respect to turbine choice open until quite late in the process. This gave them more lead way in negotiating with turbine manufacturers—but as argued before, this could also create room for a more deliberate value sensitive choice of turbines (it is not known though if any value considerations played a role in this case). Yet postponing the turbine choice meant that the project had to apply for building permits for each of the different turbine designs considered—which was a demanding administrative undertaking. One thing that the Zuidlob case could be taken to illustrate, then, is that the feasibility of a VSD approach to wind energy projects will partly depend on the broader institutional environment enabling and constraining certain processes.^16^

## A Challenge: Wind Parks as Socio-Technical Systems

The previous sections featured several ways in which institutional elements could come into play in wind park design, as ‘objects’ of design, as providing both possibilities for and constraints on design outcomes, and as either facilitating or constraining design processes. Taking a step further, it could be argued that wind parks can best not be understood as collections of technical artifacts at certain geographical locations, but rather as being integrated socio-technical systems, “engineering systems that need actors and some social/institutional infrastructure to be in place in order to perform their function” (Ottens et al. 2006, p. 135). Depending on where one draws the system’s boundaries, one may also say that wind parks are socio-technical systems which are embedded in an even larger socio-technical system—the national or even international energy system as a whole. Safeguarding critical functions within such large and complex systems is only possible if technology and institutions are well-aligned. Non-moral values such as reliability of these energy system may pose additional demands and conditions on the design of wind parks (Kunneke 2008; Künneke et al. 2010).

The challenge is that “the design of social elements […] lies largely beyond the scope of current engineering practice” (Ottens et al. 2006, p. 141). In fact, the idea of designing socio-technical systems has been challenged in the literature. Not only is it the case that “appropriate comprehensive design processes and methods are still lacking”, there is also “not even a consensus as to the prospects and limits of all-inclusive design in socio-technical systems” (Bauer and Herder 2009, p. 602). Although “design choices” in principle exist at all levels at which a socio-technical system could be defined (from small and limited to large and comprehensive), at higher levels “deliberate design decisions become less prevalent and emergent characteristics become more important” (Bauer and Herder 2009, p. 605). Furthermore, “as all purposive decisions are made in social settings” what matters is not only functional and normative design criteria, but “the process of decision making and the participating stakeholders will also influence the outcomes” (Bauer and Herder 2009, p. 606). “Many actors *within* the socio-technical system are continuously changing (redesigning) the system”, Kroes et al. (2006, p. 814) notice, “which makes the idea of ‘total design control’ problematic.” This is reinforced, they say, by the fact that the function of socio-technical systems is often contested—whereas the intended artifact function is central to engineering design. Somewhat similarly, Bauer and Herder (2009, p. 608) claim that “socio-technical design issues often pose “wicked”, poorly defined and evolving problems. It is thus not surprising that the previous section raised questions about the boundaries and scope of wind parks as potential VSD projects. The literature on Value Sensitive Design (VSD) is not going to be helpful here either, as it has been focused mainly on software and artifact design. Within VSD there has been a lack of attention for the long-term impacts of design choices and for wider socio-technical systems (Nathan et al. 2008). In that sense, the application of VSD to wind energy may also expand our understanding of the possibilities and limitations of VSD, and provide a stimulus for expanding existing methods and tools that can be applied in VSD.

Although “the possibility of a comprehensive, outcome-oriented planning [and design] process” can be questioned for socio-technical systems, so Bauer and Herder (2009, p. 625) conclude, “practical experience has generated ample evidence that design choices and planning can make a significant difference.” Wind parks are socio-technical systems at a level that is still concrete enough to expect that making this difference is indeed possible, and to pose that adopting ideas from the VSD literature may indeed be fruitful. Yet although VSD may lead to more acceptable solutions, it is unlikely that all parameters relevant for social acceptance will be within the scope of the design project in each case. Even with the most extensive way of framing a feasible VSD project within the wider socio-technical system, some specific design alternatives will not be feasible or within reach for all sorts of reasons—including institutional limitations. The scope and boundaries of a VSD project within this domain of wind energy may there itself become a topic for controversy. One question that one may ask at this point is whether wind park development should be conceptualized and studied as a *design* process (adopting methods and approaches from design studies), or whether perhaps some ‘social shaping of technology’ perspective is more useful (adopting methods and approaches from the field of science and technology studies, or STS). Insights from STS that may prove useful for the case of wind park design are for example that of the “interpretative flexibility” and the “agency” of technical artifacts. However, these ways of looking at wind park development could also be considered as complementary, as they both share the assumption that the exact materiality and shape of wind parks and turbines matters from a moral and political perspective, and is not fixed by what is scientifically ‘best’.

## Conclusion

Inspired by research showing the importance of a range of moral values—such as distributive justice and sustainability—in creating social acceptance of wind parks, this article has explored the possibility of adopting a value sensitive design (VSD) approach towards wind turbines and wind parks. Research on the social acceptance of wind energy currently treats wind parks and turbines, the central ‘objects’ towards which motivation/attitudes are directed, and which elicit certain behavioral responses of stakeholders, as ‘black boxes’. As a result, this literature provides too little guidance on what concrete design alternatives are available when one wishes to develop a wind park that strikes an acceptable balance in respecting or accommodating the key values at stake. At the same time, the literature that is available on wind park and turbine design is often too much geared towards engineers and technical aspects, providing little in-depth discussion of value issues. Or it merely discusses a single value—such as aesthetics or sustainability—rather than taking an integral approach towards values.

A VSD approach of wind parks and turbines is meant to supplement rather than replace the bottom-up, participatory process-oriented approach promoted by the current social acceptance literature. Arguably adopting an outcome-oriented, value sensitive design approach would lead to wind parks that are more *acceptable* from a value perspective, which in turn may lead to a greater actual community *acceptance* by stakeholders. Whether the latter is indeed the case remains to be seen, and will of course also depend on the process involved; Improving the acceptability of the outcome through VSD seems, at least in the short term, neither necessary nor sufficient for creating community acceptance for a project. Yet such a VSD approach may be nevertheless helpful.

It goes perhaps without saying that future research on this topic would have to be interdisciplinary (Taebi et al. 2014), drawing amongst others on the VSD literature and the STS literature. It was mentioned before that it would be useful to have more case studies looking—from a value perspective—into the design alternatives considered during a wind park development. These cases could provide input for some sort of ‘wind park VSD library’ which could become a resource for future wind park development projects. Further research should—amongst others—also look into two somewhat related challenges which were discussed in the paper, namely that of establishing the boundaries of the wind energy project to which VSD will be applied, and that of applying VSD to a socio-technical system such as a wind park. Such research would not only lead to helpful insights for the application of VSD in this domain, but it could also further our understanding of VSD itself.

Although this paper has identified gaps in the existing academic literature, this is of course done with the aim of stimulating research that will ultimately facilitate the transition to renewable energy—including wind energy. Considering the importance of this societal goal and the difficulties in achieving it, alternative approaches like the one discussed in this paper are worth considering.

## Footnotes

1 This is a claim made by the NWO-funded project “Platform wars for socially responsible smart grids: the influence of stakeholder networks and platform flexibility” (http://www.nwo.nl/onderzoek-en-resultaten/onderzoeksprojecten/26/2300178426.html, accessed June 18th 2013).

2 It is worth noting though hat according to Bidwell (2013 p. 190) “there is [still] a lack of empirical evidence for how values influence attitudes towards wind energy development.”

3 Note though that according to Wolsink (2007, p. 1193) “the variance of the landscape and scenic aspects of wind power is greater than for the other categories [of possible consequences of the application of wind energy], indicating that this is the aspect about which there is most disagreement.”

4 Other conceivable public responses mentioned by them are e.g. uncertainty, apathy, agreement, and resistance.

5 It should be noted though that power relations between stakeholders may prevent a participatory design or decision-making process leading to a fair/just outcome (a point that was already raised by the discussion on acceptance versus ‘acceptability’ in the previous section). See also e.g. the case discussed by Anderson (2013), in which a minority group with strong social capital was able to prevail in a local wind farm conflict. Another group, larger but with low social capital, was unable to bring their view on the development across and influence the process. Anderson suggests that different participatory processes are needed for both types of groups. See also Gray et al. (2005) on the difference in bargaining power between fishers and wind park developers in the case of offshore wind farms in the UK.

6 Gross (2007) extensively discusses the complexity of the relationship between ‘process fairness/justice’ and ‘outcome fairness/justice’ in relation to a wind energy project. She shows amongst others that in her case study “attitudes regarding the legitimacy of the outcome were influenced by perceptions of fairness [of the process].”

7 It may be helpful though to turn this into a hierarchy of four levels by splitting the level of value into two sub-levels, using a distinction that has amongst others been made by Rawls (1999p. 5): the distinction between the concept of a value, and the conception of a value. The concept is the general idea of a value, such as justice or sustainability. The conception is a specific interpretation or understanding of the meaning of that value.

8 See the project website at http://www.wind.psu.edu/BIWE/, accessed 29 July 2013.

9 The reason why higher turbines reduce the probability of bird collision is mentioned by Mathew and Philip (2012, p. 96): as higher towers catch more wind, they have larger rotors. And “due to the larger rotor size, bigger turbines are designed to run slower to keep the optimal tip speed ratio. […] Lower rotational speed minimizes the risk of avian mortality”.

10 These values may have further aspects or alternative interpretations not discussed so far. There may also be other consequences of this basic design choice (height/size of turbine) that have not been discussed here, but which may be relevant from a value perspective. And of course there will be other aspects of the full design that are also relevant from a value perspective.

11 A positive exception is the European project Mermaid, whose systematic and structured approach to exploring “innovative multi-purpose offshore platforms” includes the integration of wind energy. See http://www.mermaidproject.eu/ for more information on the project.

12 The author got this idea from a personal conversation with Kas Hemmes, which took place in the course of 2013.

13 The expectation that it will lead to larger community *acceptance* is in practice a motivation for developers to provide community benefits or creating community ownership (Hall et al. 2013), although there exists the risk that introducing community benefits will backlash when perceived as “buying consent” by the local population (Huber and Horbaty 2010).

14 Concerns have for example been expressed—based on recent psychological research findings - that such community benefits may erode moral motivation (Walter 2012). A recent case study indicates that people might actually be more supportive of a local wind farm initiative when it is part of a larger vision of realizing the common good (Firestone et al. 2009). Furthermore, one may question the rationale of or justification for community benefits (compensation or fairness?), and their legitimacy (Walter 2012).

15 This example is derived from a presentation by Douwe Monsma (inhabitant of the area) and Margrit Delmel (energy company Nuon), who have both worked on this project, at the Nationaal Windenergie Congres (May 16th 2013, the Netherlands). A further clarification of the example was acquired through a short e-mail exchange with Deimel, which took place in December 2013. Unfortunately, there are no articles or public reports that document or discuss the micro-siting choice or turbine choice made in this project.

16 Fortunately the recent introduction of a new piece of legislation in the Netherlands, the so-called “rijkscoördinatieregeling”, has now simplified this process by making one ministry responsible for coordinating all relevant permits and aligning the appeal procedures.
